# Gold-Nanocluster-Assisted Nanotransfer Printing Method for Metasurface Hologram Fabrication

**DOI:** 10.1038/s41598-019-38891-2

**Published:** 2019-02-28

**Authors:** Soon Hyoung Hwang, Jaebum Cho, Sohee Jeon, Hyeok-Jung Kang, Zhi-Jun Zhao, Sungjae Park, Yohan Lee, Jonghyun Lee, Mugeon Kim, Joonku Hahn, Byoungho Lee, Jun Ho Jeong, Hwi Kim, Jae Ryoun Youn

**Affiliations:** 1 Research Institute of Advanced Materials (RIAM), Department of Materials Science and Engineering, Seoul National University, Seoul, 08826 South Korea; 2 Nano-Convergence Mechanical Systems Research Division, Korea Institute of Machinery and Materials, Daejeon, 34103 South Korea; 3 Department of Electronics and Information Engineering, Korea University, Sejong, 30019 South Korea; 4 School of Electrical and Computer Engineering, Seoul National University, 1 Gwanak-ro, Seoul, 08826 Gwanak-gu South Korea; 5 School of Electronics Engineering, Kyungpook National University, Daegu, 41566 South Korea

## Abstract

Given the development of nano/microscale patterning techniques, efforts are being made to use them for fabricating metasurfaces. In particular, by using abrupt phase discontinuities, it is possible to generate holographic images from two-dimensional nanoscale-patterned metasurfaces. However, the fabrication of metasurface holograms is hindered by the high costs and long fabrication time involved, because the process requires expensive equipment such as that for electron-beam lithography. Therefore, it is difficult to realize metasurface holograms in a fast and repetitive manner. In this study, we propose a method for fabricating metasurface holograms based on the nanotransfer printing of the desired nanoscale patterns, which is assisted by Au nanoclusters, while controlling the bonding energy based on the shape of the deposited Au layer. Robust covalent bonds are formed between the Si of the adhesive used and the O of the SiO_2_ layer in order to transfer the deposited Au onto the transparent substrate quickly. It was found that the fabricated metasurface hologram coincides with the one designed by computer-generated holography. The proposed method should lead to a significant breakthrough in the fabrication of holograms based on different types of metasurfaces at a low cost in a fast, repetitive manner with various metals.

## Introduction

The design of two-dimensional nanostructured materials called metasurfaces has introduced a new paradigm in nanophotonics^1^. Metasurfaces are typically patterned to allow the modification of the amplitude or phase of a transmitted or reflected light wave by causing a discontinuity in the phase of the light that is transmitted through or reflected from them^2^.

Owing to their ability to synthesize optical fields at subwavelength thicknesses, metasurfaces have recently been used to produce meta-holograms^3–6^. The first meta-hologram was demonstrated by the Larouche group at infrared wavelengths^7^. They used H-shaped gold discs and rectangular patches to produce the meta-hologram. The effective refractive index changed with the dimensions of the metamaterial elements. As nanofabrication techniques develop further, the size of the metasurface scatterers will diminish even more, making it possible to produce meta-holograms in the visible-wavelength region. The Chen group could generate meta-holograms of different images using linearly polarized incident light^8^. The underlying principle of these meta-holograms was that the reflectance and phase distribution are functions of the length of the nanorods and the wavelength of the incident light used. The Au nanorods of four different lengths were used so that different images could be reconstructed by the linearly polarized light along the *x*- or *y*-direction. However, the shapes of these scatterers were based on a design that considered two plasmonic resonances along orthogonal directions. It is difficult to cover the entire range of phase modulation while keeping the scattering amplitude constant; this is the case regardless of the wavelength of the incident wave. To solve these problems, Huang and his colleagues demonstrated a metasurface that induced an abrupt phase change in a circularly polarized wave at visible wavelengths. The metasurface was composed of an array of metallic nanorods having the same geometry but spatially varying orientations^9^. They showed that the scattered wave is partially converted into a circularly polarized wave having the opposite handedness, with the phase difference being determined solely by the orientation of the dipole when the circularly polarized wave is incident onto a dipole antenna. In other words, 2*π*-range phase modulation could be achieved merely by changing the orientation angle of the nanorods from 0 to *π*. They applied this principle to produce holograms and demonstrated a computer-generated holography (CGH)-based hologram plate using metal nanorods^10^. Given the simplicity and ease of fabricating metasurfaces with this geometry, there have been several studies on rotating nanorods. To enhance the hologram efficiency, researchers had designed metasurfaces to achieve a high polarization conversion rate^11^. Moreover, since Chen and his colleagues demonstrated multicolored meta-holograms by integrating three plasmonic pixels that had different sizes and were designed to respond to red, green, and blue lights onto a metasurface in 2015^12^, several studies on meta-holograms that exhibit colors other than RBG have been reported^13–15^. Furthermore, there have also been studies on the reconstruction of the target image by controlling the orthogonal polarization of the incident wave^16,17^.

However, although several studies have investigated metasurface holograms in detail, the fabrication of metasurface CGH holograms remains a costly and time-consuming process because conventional metasurface holograms are realized by direct writing on the substrate using electron-beam lithography and lift-off processes, or focused ion beam (FIB) methods^10,11,14^. Therefore, it is difficult to produce metasurface holograms repetitively and quickly at a low cost with various materials. In view of these facts, the nanotransfer printing method can be an effective solution because it allows for the material deposited on the polymer stamp, which is a replica of a nanopatterned Si master, to be transferred onto a substrate. In this way, the nanopattern can be reproduced on a substrate in a fast and repetitive manner with various materials. In our previous work, optical elements were successfully fabricated via nanotransfer lithography using a deeper Si master composed of a line and hole pattern^18^. However, in the case of metasurface holograms, which can include nanoslits or nanorods, a high-resolution pattern with varying orientation angles is required. Therefore, it is difficult to fabricate a Si master with deeper features while maintaining the high resolution of the nanoslit or nanorod pattern with varying orientation angles. For this reason, the conventional nanotransfer printing method for fabricating high-resolution metasurface holograms with an angular nanopattern but Si a master with a low depth cannot be applied because the depositions on the top and in the trenches are connected to each other, thus forming a corrugated film. Additionally, undesired nanostructures could be transferred from the polymer stamp to the substrate. To address these challenges, in this study, we provide a new nanotransfer printing method called Gold-nanocluster-assisted nanotransfer printing (NCNP) to fabricate both nanoslit- and nanorod-type metasurface holograms using Au, Ag, and Al. This method involves forming Au nanoclusters on the polymer stamp to keep the deposited materials disconnected and to control the bonding energy between the interfaces of layers and the polymer stamp based on the state and shape of the deposited materials. This method can prevent the deposited materials on the top and in the trenches of the polymer stamp from connecting, and it can control the bonding energy in order to transfer the desired nanopatterns. The deposited materials were then transferred onto the substrate using the roll-to-plate process by quickly forming robust covalent bonds between the Si of the adhesive and the O of the deposited materials. With this method, for the first time, a metasurface hologram with a continuously varying orientation angle for inducing an abrupt phase change was fabricated successfully using the nanotransfer method in a simple and low-cost manner.

## Results and Discussion

### Design of a nanoslit metasurface hologram

**Figure 1:**
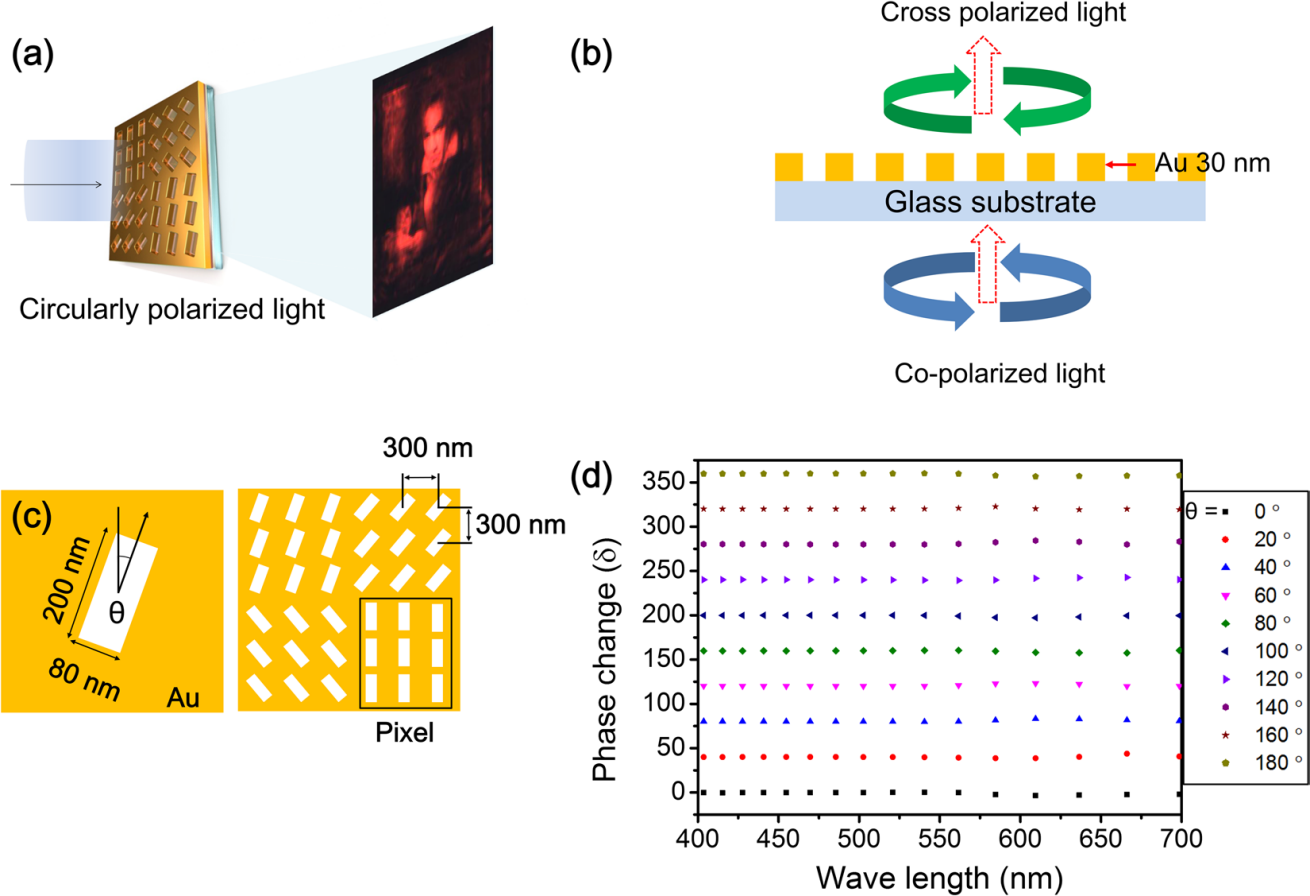
Schematic diagram of a nanoslit metasurface hologram. (**a**) Illustration of a nanoslit metasurface hologram of Ludwig van Beethoven (original painting by Joseph Karl Stieler in 1820) produced by a nanoslit metasurface and, which was designed by using a CGH technique. (**b**) The principle on which nanoslit metasurfaces is based. When circularly polarized light is incident on a nanoslit metasurface, its direction becomes opposite because of a phase change. (**c**) Illustration of a 3 × 3 array of unit pixels on a nanoslit metasurface with; the dimensions, angle, and pitch are shown. (**d**) Phase change (δ) of a cross-polarized light by designed pixel with a change in the orientation angle (θ), as calculated by an FDTD simulation.

To produce the metasurface hologram, we induced abrupt local phase changes by exploiting the geometric phase called the Pancharatnam–Berry phase^19–21^. As per the Pancharatnam–Berry phase, a phase change can only be induced by space-variant polarization manipulations, in contrast to the case for diffractive and refractive elements^14,19–21^, such as angular nanoslits or nanorods. In other words, when a coherent electromagnetic plane wave source is incident on nanoslits or nanorods, the transmitted wave is also planar but exhibits a phase shift, which originates from the change in the angle between the primary axis of the nanostructure and the polarization direction of the source wave. In the case of a linear polarized plane source, the amplitude of the transmitted wave also changes, in addition to its phase. Therefore, if circularly polarized light (i.e., a co-polarized wave) is incident on nanoslits or nanorods with an orientation angle, only the geometric phase influences on the transmitted wavefront (i.e., the cross-polarized wave)^21^. On this basis, we fabricated a nanopatterned metasurface hologram in order to induce an abrupt phase change and control the wavefront^9,10^ in the visible range. As depicted in Fig. 1a,b, when the co-polarized wave field was incident on the metasurface with angular nanoslits, a part of the co-polarized field was converted into a cross-polarized field^9,17^.

The analytic expression for the transmitted electric field in terms of the co-polarized field ([1*i*]^*T*^) and the cross-polarized field ([1−*i*]^*T*^) with the Jones’ matrix is as follows:

1 $$(\begin{array}{c}{E}_{x}\\ {E}_{y}\end{array})={\rm{a}}(\begin{array}{c}1\\ i\end{array})+{\rm{b}}{e}^{i\delta }(\begin{array}{c}1\\ -i\end{array})$$

Here, a and b are complex amplitudes with the same angle and δ is the geometric phase of cross-polarization, which is related to the angle θ between the primary axis of a nanoslit and the orientation axis (x) by δ = 2θ^14,17,19,21^, as can also be seen from the simulation result in Fig. 1d. Figure 1c shows the metasurface designed using the Au nanoslits. The efficiency of polarization is determined by the shape and material of the formed nanostructures^20^. Each slit was 200 × 80 nm and had an angular orientation θ of 20° (stepwise) as modeled using the CGH algorithm. The pixel arrays consisted of 3 × 3 rectangular nanoslits with a pitch of 300 nm for robust phase consistency. The thickness of the Au nanoslits on the glass substrate was 30 nm.

Figure 1d shows the numerical simulation results for the phase change of cross-polarized light, as determined using FDTD method and the designed single pixel (3 × 3 nanoslits). A constant phase change can be observed in the visible range (400–700 nm), which confirmed the controllability on the local phase of the designed nanoslit-based pixel.

### Design of a nanoslit metasurface hologram using CGH

**Figure 2:**
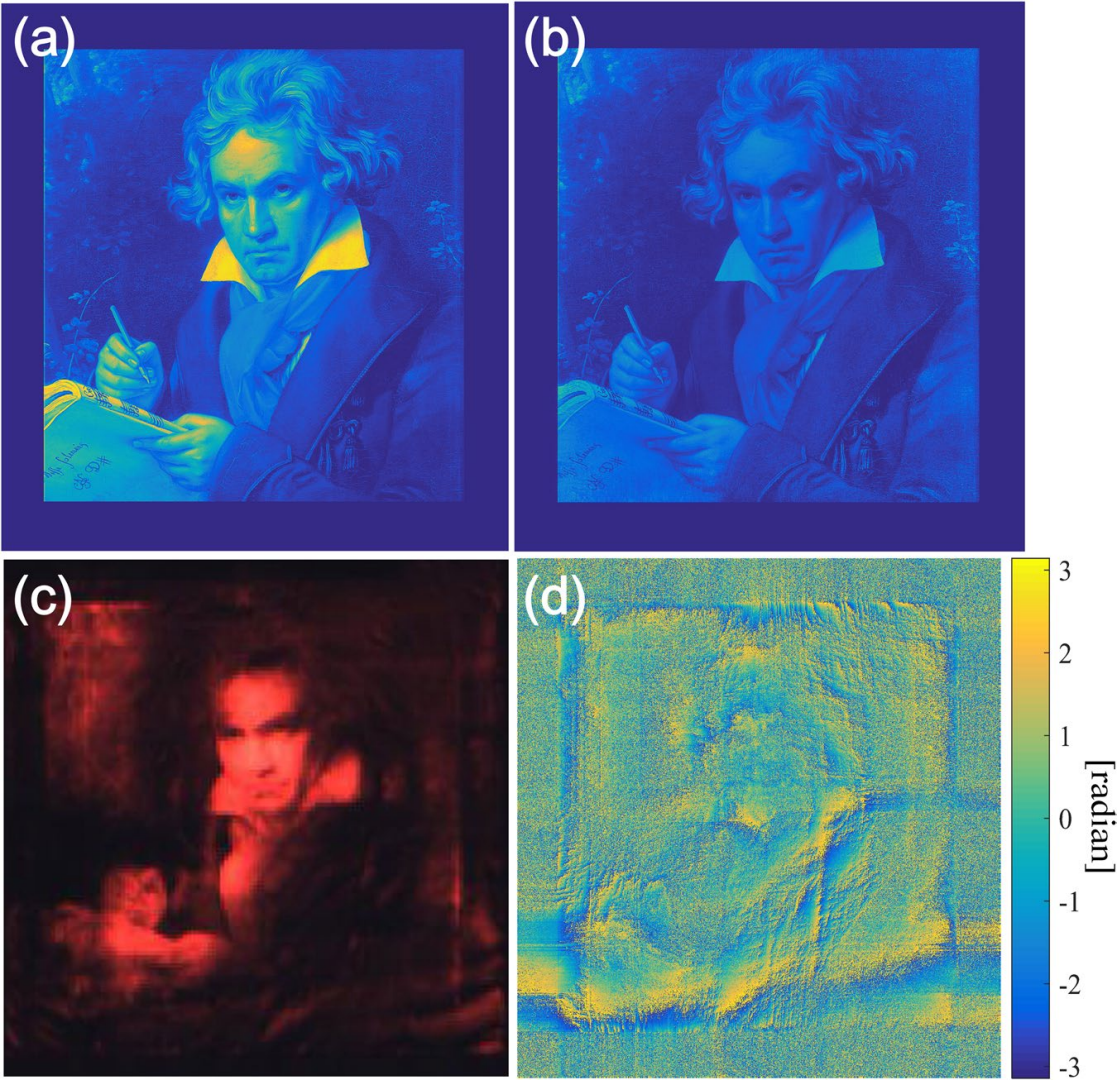
Simulation and experimental results for a nanoslit metasurface hologram. (**a**) Target image. (**b**) Numerically reconstructed profile from the calculated CGH hologram. (**c**) Experimentally obtained hologram image from a nanoslit metasurface. (**d**) Phase profile of a phase-only CGH.

Figure 2 shows the target and calculated CGH hologram images as well as the experimental results for the angular nanoslit metasurface hologram. The iterative Fourier transform algorithm (IFTA) was used to minimize the error in the phase-only CGH hologram^22^. The IFTA is an iterative algorithm for minimizing the amplitude error of holographic images and provides the phase profile of the image in terms of the degrees of freedom because only the intensity (amplitude) of the light wave can be measured. The location of the target image was set just 1 mm above the meta-hologram. The angular spectrum method (ASM) based on scalar diffraction theory was used to calculate the propagated light field. Since the inverse of the ASM is well defined, the Fourier transform and inverse Fourier transform in the IFTA can be replaced by the ASM. We set the initial profile on the metasurface to be constant. Then, the 1-mm propagated field was calculated by the ASM. If the propagated field is $u(x,\,y)\cdot \exp \{{\rm{j}}\varphi (x,\,y)\}$, *u*(*x*, *y*) is replaced by $\sqrt{I(x,\,y)}$, while *I*(*x*, *y*) is the target image, leaving the exponential term as a degree of freedom. Then, $\sqrt{I(x,\,y)}\cdot \exp \{{\rm{j}}\varphi (x,\,y)\}$ is back-propagated to the initial metasurface plane by the ASM. Since the metasurface can modulate only the phase profile, the amplitude of the back-propagated field should be 1. After the amplitude is set to 1, the field is propagated to the image plane again.

This process loop is repeated until the output image matches the criterion. As can be seen in Fig. 2a, the target image is zero-padded in consideration of the diffraction angle in order to prevent an aliasing error. The ASM is calculated by the discrete Fourier transform (DFT), and the periodic property of the DFT can cause an aliasing error without the extension of the computation grid. The total resolution of the CGH hologram was 5001 × 5001 and its size was approximately 4.5 mm × 4.5 mm. The CGH hologram was optimized for a wavelength of 660 nm.

### Fabrication of a nanoslit metasurface hologram by NCNP

**Figure 3:**
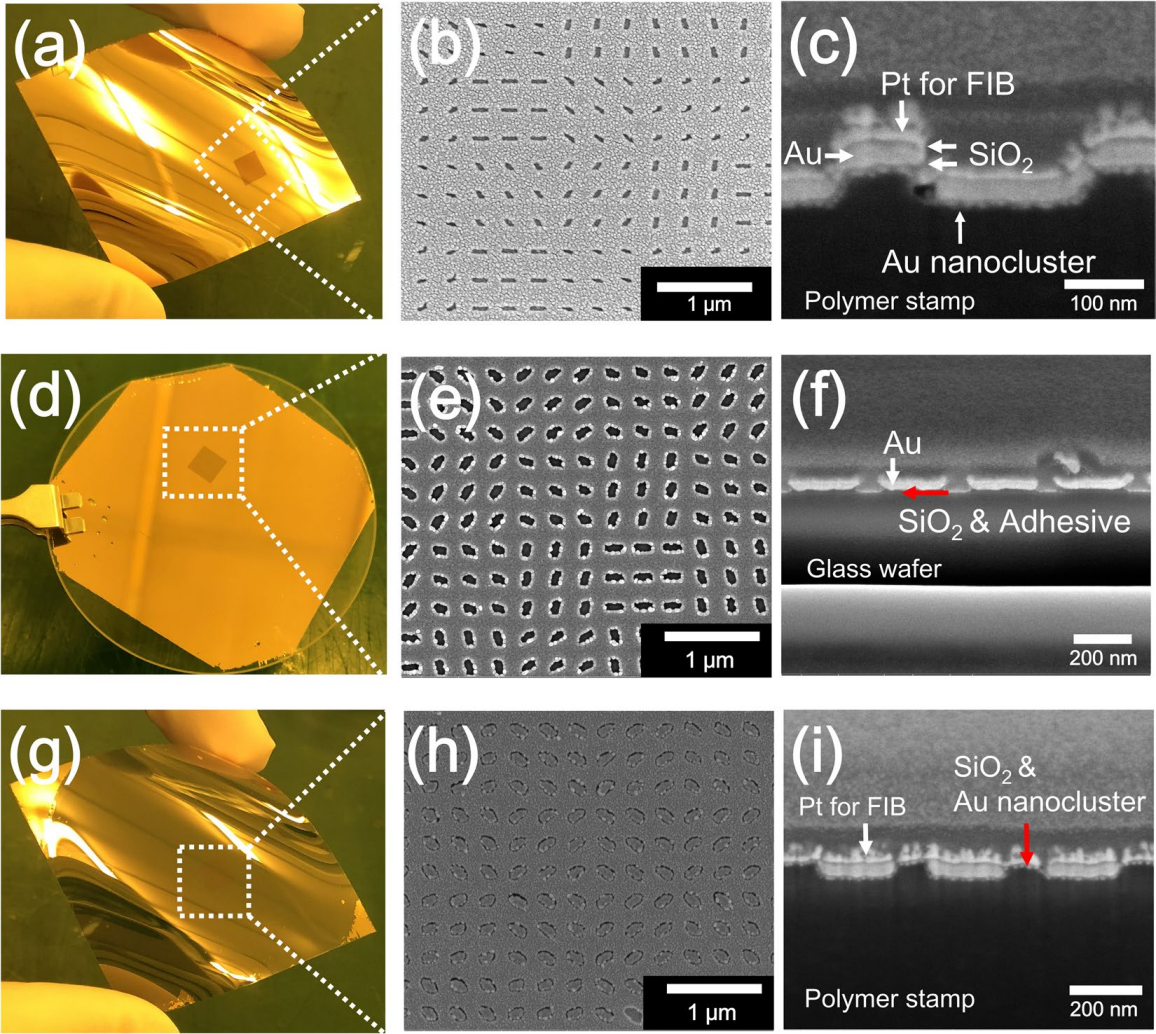
Procedure for Au nanocluster-assisted nanotransfer printing. (**a**) Si master with angular nanoslits fabricated by e-beam lithography. (**b**) Replication of angular nanoslits in the Si master using a UV-curable imprint resin and PET film. (**c**) Peeling of replicating polymer stamp with PET film after UV curing process. (**d,e**) Replication of angular nanoslits by pillars of replicating polymer stamp fabricated in (**c**). (**f**–**i**) TEM images of Au and SiO_2_ layers consecutively deposited on polymer stamp to form nanoclusters and thin film with angular nanoslits. (**j**) Prepared flexible polymer stamp with four alternating layers of Au and SiO_2_. (**k**) Nanotransfer printing using a roll-to-place system on adhesive-coated glass substrate to transfer only two layers and removal of flexible polymer stamp with remaining two layers. (**l**) Deposited SiO_2_ and Au angular nanopatterns on a polymer stamp transferred to a glass substrate, which resulted in a metasurface hologram.

We fabricated a metasurface hologram using the two previously mentioned primary techniques. First, we deposited a 5-nm-thick Au layer (denoted as Au nanoclusters) using an e-beam evaporator to form Au nanoclusters on the polymer stamp (Fig. 3f) in order to prevent connections between the deposited materials on top and in the trenches of the polymer stamp and to control the bonding energy between the layers based on their shape. Next, we deposited a 5-nm-thick SiO_2_ layer (denoted as the intermediate layer), a 30-nm-thick Au (denoted as the Au thin film), and another 5-nm-thick SiO_2_ layer (denoted as the outermost layer) (Fig. 3i) in order to transfer the Au thin film with the angular nanoslits onto an adhesive-coated glass substrate based on the formation of covalent bonds between the outermost layer of SiO_2_ and adhesive. This ensured that the deposited Au thin film and SiO_2_ layers were both successfully transferred onto the substrate (Fig. 3i–l)^18^.

**Figure 4:**
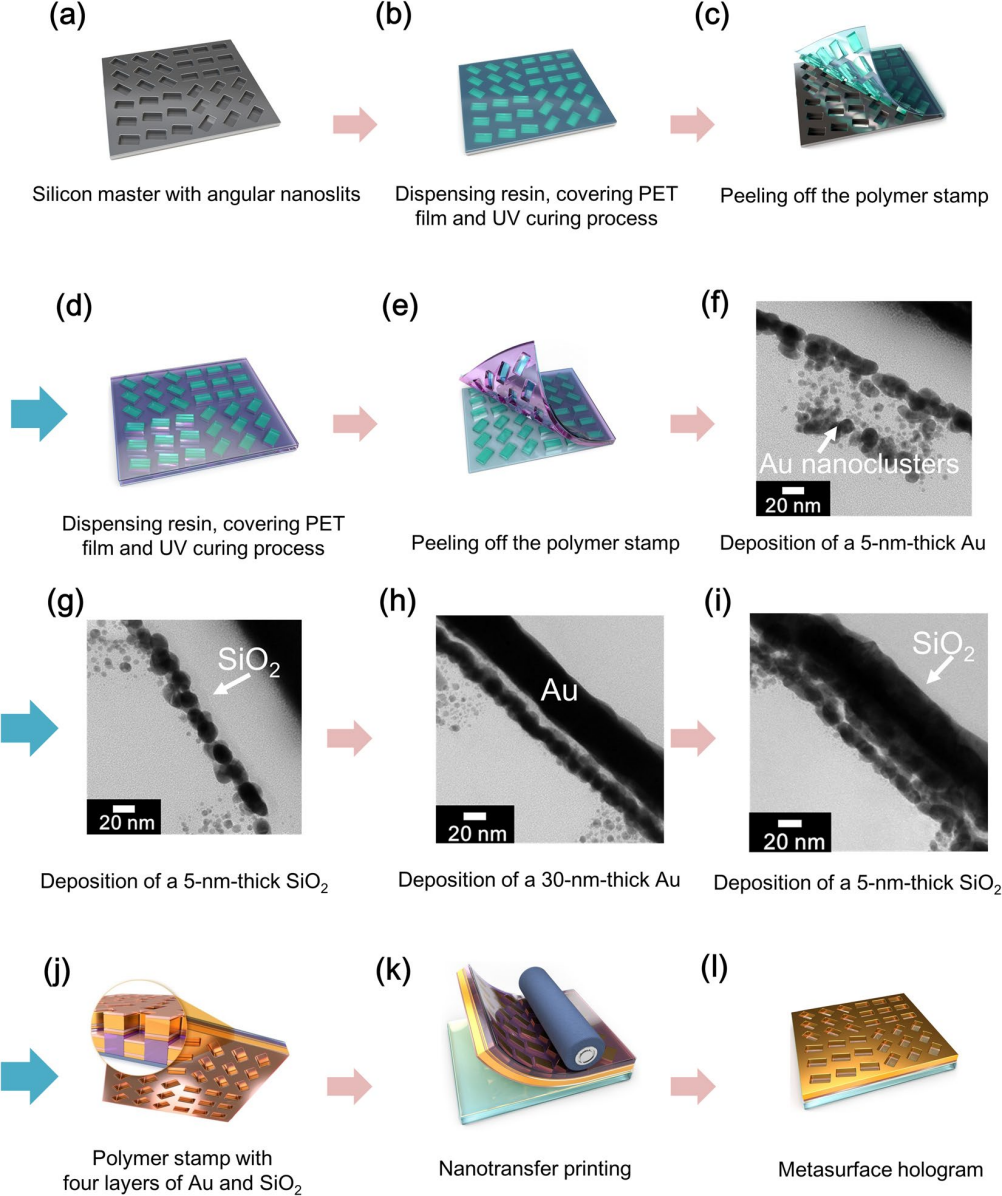
Images of the fabricated angular nanoslit metasurface. (**a**) Photograph of the flexible polymer stamp with four deposited layers of Au and SiO_2_. (**b,c**) FIB images of the surface and cross-section of the Au and SiO_2_ layers deposited on the polymer stamp. (**d**) Nanoslit metasurface fabricated by transferring Au and SiO_2_ layers onto an adhesive-coated 2-inch glass substrate. (**e,f**) FIB images of the surface and cross-section of Au and SiO_2_ layers transferred onto the glass substrate. (**g**) Photograph of the flexible polymer stamp after transfer of the Au and SiO_2_ layers were transferred. (**h,i**) FIB images of the surface and cross-section of the polymer stamp after transfer of Au and SiO_2_ layers onto the glass substrate.

Figure 3 shows the procedure for fabricating the nanoslit metasurface hologram by NCNP. The pattern on the flexible polymer stamp was replicated using the Si master (Fig. 3a–e). Then, four layers of Au and SiO_2_ were deposited on the polymer stamp (Fig. 3f–j). When only two layers, namely, the metal film and outermost SiO_2_ layer, were deposited on a polymer stamp without nanoclusters, as shown in Supplementary Fig. S1, it resulted in two cases. In the first case, as shown in Supplementary Fig. S1(b), only the SiO_2_ layer was transferred onto the adhesive-coated substrate while the metal film remained attached to the polymer stamp. In the second case, the metal thin film deposited on the top and in the trenches of the polymer stamp were connected and transferred together onto the substrate, as shown in Supplementary Fig. S3. Thus, it was essential to keep the metal thin film layer disconnected and control the bonding energy between the deposited materials and the polymer stamp, as this enabled successfully transferring the desired layers of the deposited materials from the polymer stamp onto the substrate to fabricate the nanoslit-type metasurface hologram. Therefore, we deposited Au in the form of Au nanoclusters by e-beam evaporation (Figs 3f and 4, Supplementary Fig. S1(c), and Supplementary Fig. S4), since a 5-nm-thick Au layer is not thick enough to be a thin film. Then, an intermediate SiO_2_ layer was deposited on the polymer stamp, followed by the Au thin film and outermost SiO_2_ layers (Fig. 3f–j). As a result, the outermost SiO_2_ layer formed covalent bonds with the spin-coated adhesive layer on the glass substrate, and of the four layers, only the Au thin film and outermost SiO_2_ layer were transferred from the replicating polymer stamp through covalent bonding between the Si of the adhesive layer and the O of the SiO_2_ layer. Owing to the formation of robust covalent bonds, the Au thin film and outermost SiO_2_ layer could be transferred quickly onto the adhesive-coated substrate (see the Experimental Section). The bonding strength between the deposited materials was lower than that between the SiO_2_ layer and the adhesive layer. Finally, only the two outermost layers, namely, the Au thin film and outermost SiO_2_ layer, could be transferred onto the substrate with ease, even though they consisted of multiple metal and SiO_2_ layers.

The TEM images in Fig. 3f–j and Supplementary Fig. S4 show the Au and SiO_2_ layers deposited alternately on the polymer stamp. It can be seen clearly from Fig. 3f that Au nanoclusters were formed. This was the case until three additional SiO_2_, Au, and SiO_2_ layers were deposited on the polymer stamp, with a SiO_2_ layer in thin-film form being present between the Au nanoclusters and the Au thin film to prevent contact between the two and ensure that the shape of the nanoclusters was maintained. The energy dispersive spectroscopy (EDS) results shown in the inset of Supplementary Fig. S4 confirmed that the four layers were distinct.

The photographs in Fig. 4a and the FIB images in Fig. 4b,c show the flexible polymer stamp (dimensions of 4.5 × 4.5 mm) with the four deposited layers of Au and SiO_2_. Both the Au nanoclusters and the intermediate SiO_2_ layer prevented the formation of connections of the Au thin film because its cross-sectional image is different from that of the Au thin film in Supplementary Fig. S1a. The photograph in Fig. 4d and the FIB images in Fig. 4e,f show the metasurface hologram fabricated on a 2-inch glass wafer by NCNP. The photograph in Fig. 4g and the FIB images in Fig. 4h,i show the flexible polymer stamp after the nanotransfer printing process; the images shown are surface and cross-sectional FIB images. It is evident that the Au nanoslit patterns and the outermost SiO_2_ layer were transferred onto the glass substrate and that a nanoslit metasurface hologram was fabricated successfully. It can also be seen that only two layers of Au and SiO_2_ were transferred onto the glass substrate (see Fig. 4f,i). As shown in Fig. 4i, the Au nanoclusters and the intermediate SiO_2_ layer remained on the top layer of the polymer stamp, while the four deposited layers of Au and SiO_2_ were present on the trenches after the nanotransfer printing process. The fabricated metasurface hologram sample (Fig. 4f) was analyzed using the setup shown in Supplementary Fig. S2, and it was observed that the obtained image was consistent with the simulation results, as shown in Figs 2 and S7.

**Figure 5:**
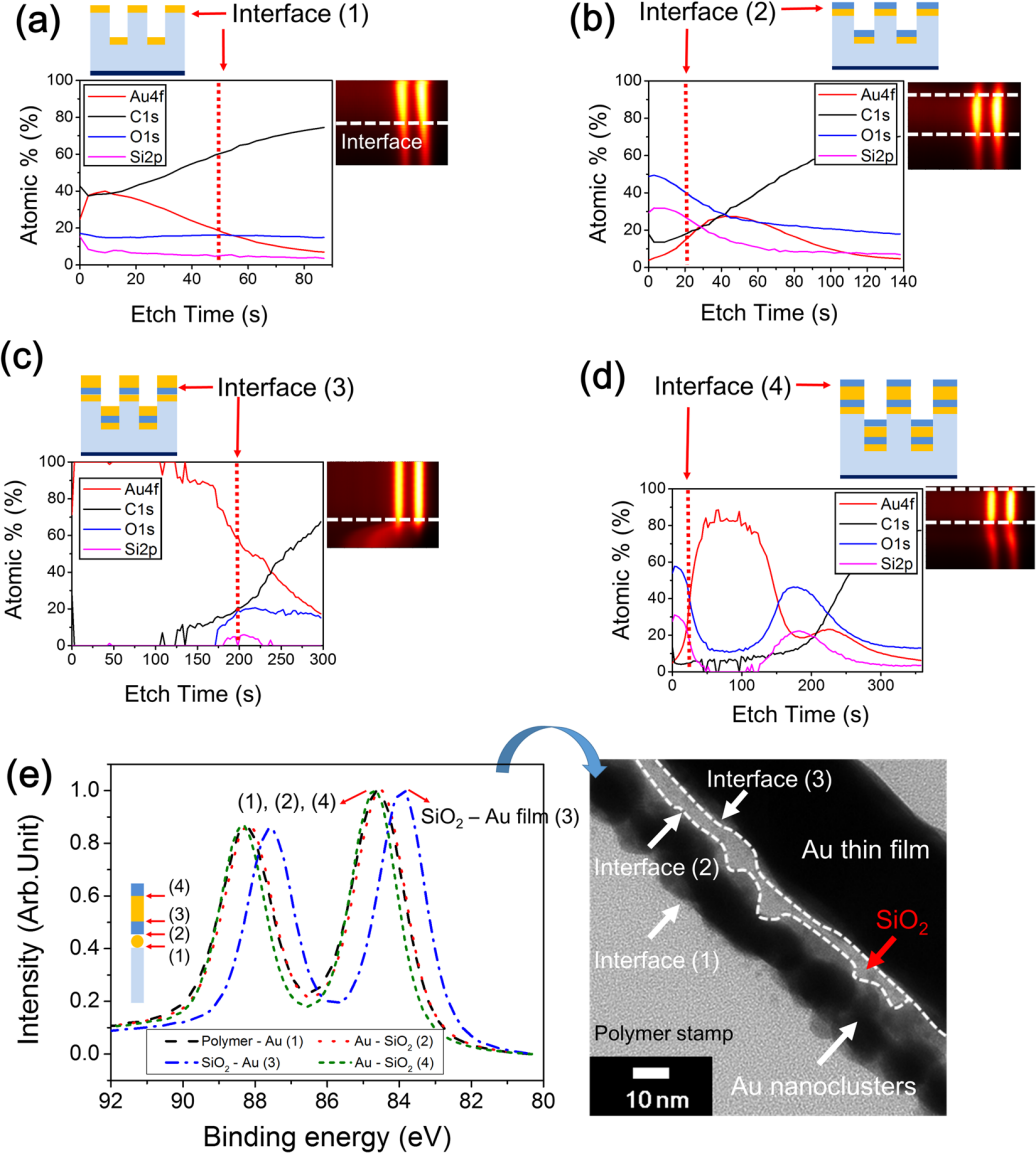
XPS spectra showing that the composition (at%) for different etch times depends on the deposited materials and their thicknesses. (**a**) XPS spectra showing the composition (at%) of a polymer stamp with Au nanoclusters. (**b**) XPS spectra showing the composition (at%) of a polymer stamp with Au nanoclusters and intermediate SiO_2_ layer. (**c**) XPS spectra showing the composition (at%) of a polymer stamp with Au nanoclusters, intermediate SiO_2_ layer, and Au thin film (**d**) XPS spectra showing the composition (at%) of a polymer stamp with Au nanoclusters, intermediate SiO_2_ layer, Au thin film, and outermost SiO_2_ layer (insets show the binding energy of Au for the corresponding samples) (**e**) Normalized XPS spectrum of the Au binding energy at each interface. (The inset shows the interfaces between materials, while the TEM image shows various interfaces).

To elucidate the mechanism responsible for the results shown in Fig. 4, X-ray photoelectron spectroscopy (XPS) measurements were conducted to compare the binding energies of Au at different interfaces. Five different samples were prepared (see Fig. 5 and Supplementary Fig. S5), namely, a replicating polymer stamp without any deposited materials; a polymer stamp with Au nanoclusters; a polymer stamp with Au nanoclusters and an intermediate SiO_2_ layer; a polymer stamp with Au nanoclusters, the intermediate SiO_2_ layer, and an Au thin film; and a polymer stamp with Au nanoclusters, the intermediate SiO_2_ layer, an Au thin film, and an outermost SiO_2_ layer. Each sample was etched to dimensions of 200 μm × 200 μm by XPS (Supplementary Fig. S5), in order to determine the binding energy of Au at each interface. The results obtained are shown in Fig. 5e. The numbers next to the inset illustration in Fig. 5e represent the interfaces. In other words, (1) represents the interface between the polymer stamp and the Au nanoclusters, (2) represents the interface between the Au nanoclusters and the intermediate SiO_2_ layer, (3) represents the interface between the intermediate SiO_2_ layer and the Au thin film, and (4) represents the interface between the Au thin film and the outermost SiO_2_ layer. It was expected that the Au nanoclusters would affect the binding energy, as it is known that the XPS peaks shift to a higher binding energy when the metal present is in the form of clusters instead of a bulk phase^23,24^.

The 5-nm-thick Au layer deposited on the polymer stamp was in the form of nanoclusters, while the 30-nm-thick Au layer was in the thin-film form, as shown in Figs S4 and 4c. Owing to these differences in the shape of the layers, there was a shift in the binding energies. As can be seen from the Au binding energy spectrum of each interface in Fig. 5e, the binding energies of interfaces (1), (2), and (4) are similar. Further, interfaces (1), (2), and (4) have higher binding energies than that of interface (3) (Fig. 5e), with the difference being approximately 0.8 eV. Further, it is likely that the detachment during the transfer process occurs at interface (3). This is because, at interfaces (1), (2), and (4), the Au present is in the form of Au_x_–Si_1−x_, and the binding energies of these interfaces are 84.63, 84.47, and 84.63 eV, respectively; these are higher than the binding energies of interface (3) (83.83 eV)^25,26^. These results suggest that, at interfaces (1), (2) and (4), an Au_x_–Si_1−x_ type of state exists, while at interface (3), an Au–Au type of state exists. This is also confirmed by the Si/Au ratio, as shown in Table 1 and Supplementary Fig. S6. On comparing the Si/Au ratios for the various interfaces (i.e., for interfaces (1), (2), (3), and (4)), it was determined that the Si/Au ratios for interfaces (1), (2), and (4) are higher than those for interface (3). This further confirmed that the Au exists as Au_x_–Si_1−x_ at interfaces (1), (2), and (4), owing to which there is a shift in the binding energy. Thus, the detachment occurred at interface (3) because of the presence of Au in only the Au–Au state and not the Au_x_–Si_1−x_ state, owing to which the binding energy of (3) was the lowest. This allowed the two outermost layers to be transferred onto the glass substrate.

**Table 1:** Si/Au ratio values and binding energies at interfaces.

Interface	Si/Au × 100	Binding energy (eV)
(1)	1.256	84.63
(2)	4.150	84.47
(3)	0.5590	83.83
(4)	3.842	84.63

**Figure 6:**
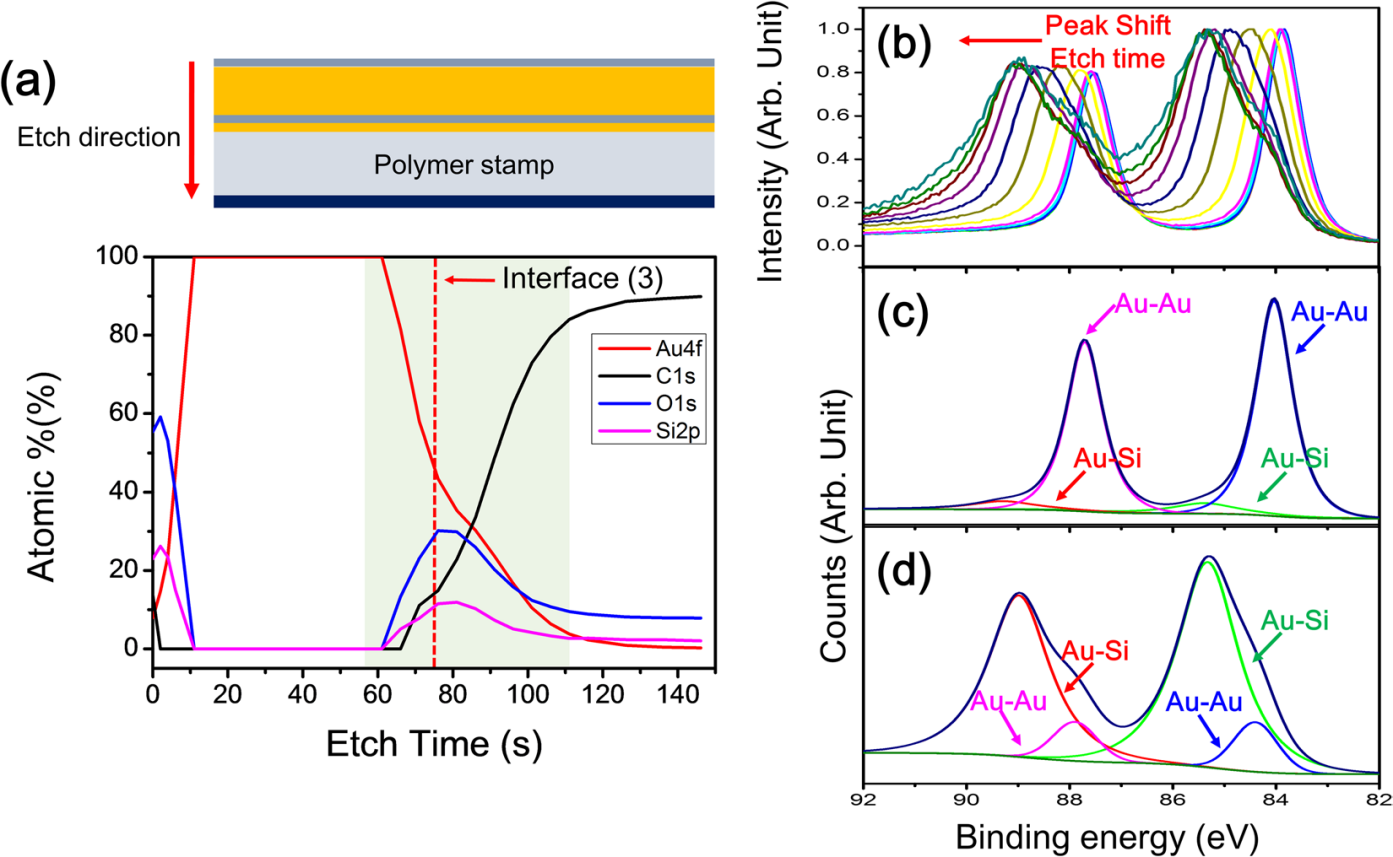
XPS spectra using a single sample without a pattern. (**a**) Illustration of the prepared sample and XPS spectra showing the composition (at%) of the sample. (**b**) Normalized XPS spectrum of the Au binding energy in the vicinity of interface (3) in the green area in (**a**). (**c**) Deconvoluted XPS spectra for the Au binding energy at interface (3). (**d**) Deconvoluted XPS spectra for Au binding energy near interface (2).

To prove that the binding energy shift is not caused by charging effects, we conduct a detailed XPS analysis. We used a single sample with no pattern because the beam spot size (200 μm × 200 μm) is larger than the pattern pixel size. Thus, we prepared a polymer film without a pattern and deposited the Au nanoclusters, intermediate SiO_2_ layer, Au thin film, and outermost SiO_2_ layer, as shown in Fig. 6(a), which exhibits the composition (at%) of Au, C, O, and Si. Figure 6(b) shows how the XPS spectrum of the normalized Au binding energy evolves with etching time in the vicinity of interface (3) (green area in Fig. 6(a)). As shown in Fig. 6(b), as the etching time increases, the Au binding energy peaks shift toward higher binding energies because as the etching time and depth increase, the Au–Au state within the Au thin film and at interface (3) change into the Au_x_–Si_1−x_ state^27–31^. For a more detailed analysis, we deconvoluted peaks at 2 points along interface (3) and near interface (2), as shown in Fig. 6(c,d). As shown in Fig. 6(c), most of the binding energy can be attributed to the Au–Au state, and the binding energy of Au4f_7_ is 84.04 eV. However, in Fig. 6(d), the binding energy of Au is divided into Au–Au and Au_x_–Si_1−x_ states, and the intensity of the Au_x_–Si_1−x_ peak is much higher than that of Au–Au. Additionally, as shown in Fig. 6(d), the Au binding energy peaks shift to higher binding energies, and the binding energy of Au4f_7_ is 84.40 eV. The results from Fig. 6 demonstrate that Au nanoclusters increase the binding energy of Au, thus verifying that Au exists near interface (2) in the Au_x_–Si_1−x_ state.

**Figure 7:**
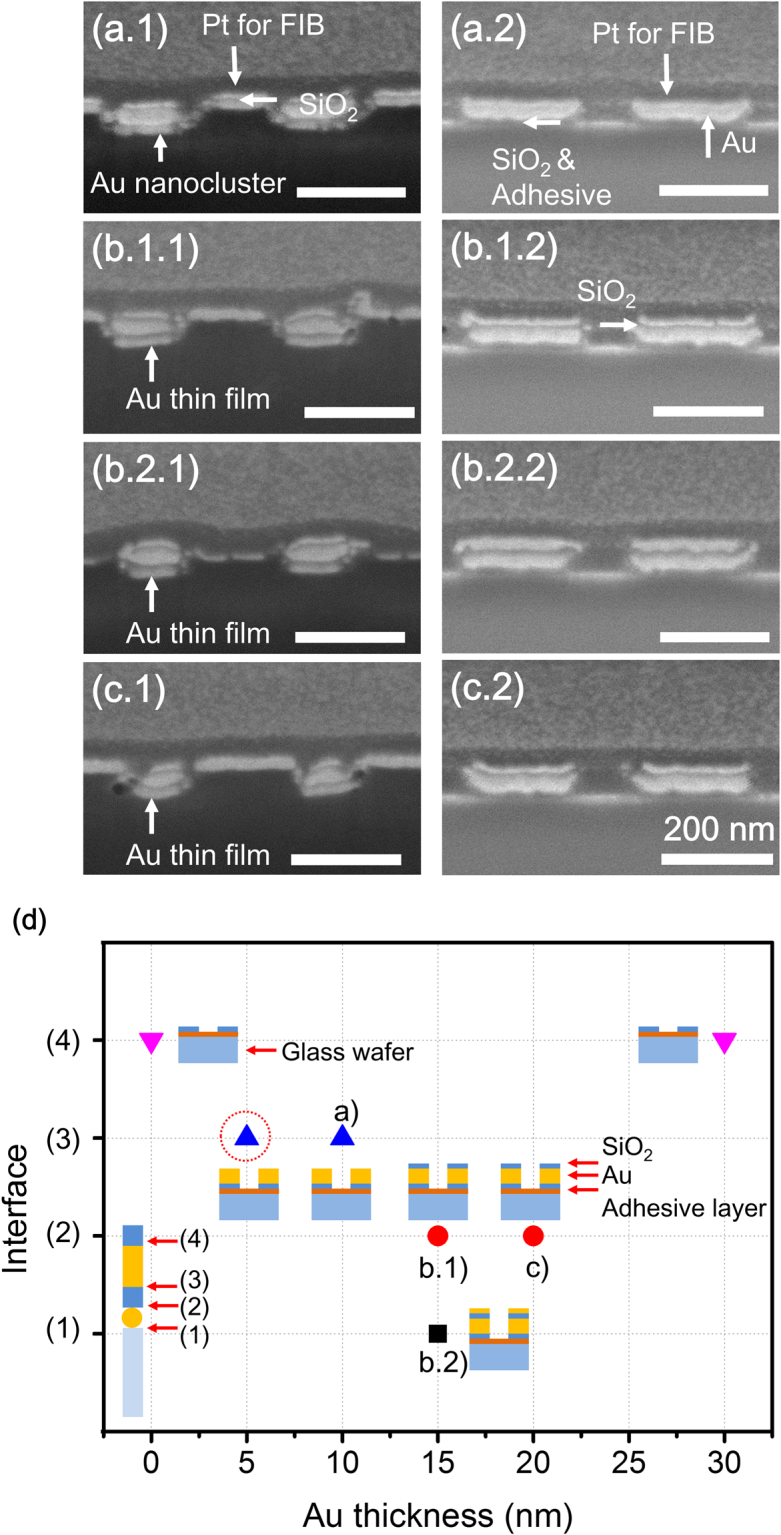
Cross-sectional FIB images of the polymer stamp and glass substrate after the nanotransfer process with (**a**) 10-nm-thick Au, intermediate SiO_2_ layer, Au thin film and outermost SiO_2_ layer, (**b**) 15-nm-thick Au, intermediate SiO_2_ layer, Au thin film, and outermost SiO_2_ layer, and (**c**) 20-nm-thick Au, intermediate SiO_2_ layer, Au thin film, and outermost SiO_2_ layer deposited on the polymer stamp. (All scale bars represent 200 nm). (**d**) Summary of results obtained after the nanotransfer process for different thicknesses of Au layers on a polymer stamp. The illustrations in the graph show the layers of materials transferred onto the adhesive-coated glass substrate in various cases. Red dotted circle indicates optimized conditions for NCNP.

Figure 7 shows the effect of the thickness and shape of the Au nanostructures on the polymer stamp for the nanotransfer process for Au layer thicknesses of 10, 15, and 20 nm instead of 5 nm. We prepared three types of specimens with a layer of Au of varying thickness (namely, 10, 15, or 20 nm), an intermediate SiO_2_ layer, an Au thin film, and an outermost SiO_2_ layer, as shown in Fig. 7a–c, respectively. We then used these three specimens to transfer patterns onto adhesive-coated substrates. The cross-sectional FIB images in Fig. 7 show the results of the transfer process and the polymer stamp after the nanotransfer printing process. Figure 7a shows that, when Au was deposited in the form of a 10-nm-thick layer, Au nanoclusters formed, although not in the same amount as in the case of the 5-nm-thick layer. Therefore, the two outermost Au and SiO_2_ layers were transferred onto the substrate (Fig. 7a2), while the remaining two Au and SiO_2_ layers on the top and the four layers in the trenches of the polymer stamp remained in place (Fig. 7a1). On the other hand, when the Au layer was deposited in thicknesses of 15 nm and 20 nm, after nanotransfer process, either three layers, namely, the intermediate SiO_2_ layer, Au thin film, and outermost SiO_2_ layer, were transferred onto the substrate (Fig. 7b1.1,1.2 and c1,c2), or all four Au and SiO_2_ layers were transferred onto the substrate (Fig. 7b2.1,2.2). This was because, as the thickness of the Au layer was increased, an Au film was formed instead of nanoclusters. From these results, it can be concluded that the bonding energies at the interfaces were similar. Thus, either three layers of SiO_2_ and Au or all four layers of SiO_2_ and Au were transferred onto the substrate, as verified by FIB images of the polymer stamp obtained after the nanotransfer process. It was also verified that, when the intermediate SiO_2_ layer, Au thin film, and outermost SiO_2_ layer were deposited on the polymer stamp without the first Au nanoclusters, or when two layers of Au and SiO_2_ were deposited on the polymer stamp, the result was the same in that only one layer, namely, the outermost SiO_2_ layer, was transferred onto the substrate. These results are summarized in Fig. 7d, where the x- and y-axes indicate the deposited Au thickness on the polymer stamp and the detached interface of the deposited materials on the polymer stamp, respectively. Thus, it was confirmed that the formation of nanoclusters is critical for ensuring that only the required layers of the metasurface hologram are transferred to the substrate. Finally, it can be seen from Fig. 7d that the layers to be transferred from the polymer stamp could be selected by controlling the bonding energy based on the shape of the deposited Au.

In addition, we successfully fabricated not only metasurfaces with an inverse design but also metasurfaces made of various metals (Au, Ag, and Al) using the same silicon master (Supplementary Information Fig. S8). Thus, the NCNP method can be applied to fabricate high-resolution nanopatterns with various materials.

## Conclusions

We successfully fabricated nanoslit metasurface hologram using a nanotransfer printing method by preventing the deposited materials on the top and in the trenches of the polymer stamp from connecting and controlling the binding energy of Au by forming nanoclusters. FDTD simulations were performed to confirm that the proposed nanoslit metasurface induced an abrupt phase delay in the incident light. Based on the simulation results, we designed 3 × 3 arrays of unit pixels for the angular nanoslit metasurface by CGH. The nanoslit metasurface hologram, which was based on nanoslits whose angle varied continuously, was fabricated by the NCNP method. The observed image was coincident with the CGH simulation image. We kept the deposited materials disconnected and controlled the binding energy by forming Au nanoclusters on the polymer stamp in order to transfer the desired nanopatterns. Among the four layers deposited on the polymer stamp, the Au nanoslit patterns and the outermost SiO_2_ layer could be transferred onto a glass substrate through the formation of robust covalent bonds between the outermost SiO_2_ layer and the adhesive layer and by controlling the bonding energy at the interfaces between the intermediate SiO_2_ layer and Au thin film. TEM and XPS analyses verified that the Au binding energy was higher when Au was deposited on the polymer stamp in the shape of nanoclusters instead of in the thin film form. In addition, by varying the thickness of the Au layer deposited on the polymer stamp, it was confirmed that nanocluster formation is essential for the transfer of the desired layers and the successful fabrication of a metasurface hologram. Additionally, it was demonstrated that both nanoslit- and nanorod-type metasurface holograms made of Au, Ag, and Al were successfully fabricated by NCNP. Finally, it should be possible to fabricate not only metasurface holograms but also any nanopatterned metasurface with various materials using the proposed method in a rapid and repetitive manner at a low cost.

## Methods

### Numerical simulations

To verify phase modulation by the designed nanoslit antennas, we performed numerical simulations using the finite difference time domain (FDTD) method as implemented in the Lumerical FDTD solutions software package. A circularly polarized plane source was made to be incident on a designed pixel (3 × 3 rectangular structure) with perfect matching layers imposed in all the in-plane axes (x- and y-axes) in order to evaluate the independent operation of the pixel. The Au was modeled using the data reported by Palik^32^ while using an imaginary weight factor of 6 for better material modeling in the FDTD method in the visible range.

### Measurement of metasurface hologram

Supplementary Fig. S2 shows the setup used for measuring the properties of the produced hologram. Fiber-coupled light-emitting diodes (LEDs) with central wavelengths of 660 nm (red), 530 nm (green), 470 nm (blue), and white LEDs were used as the light source. The light source was made spatially coherent by making the light pass through an objective lens and then a spatial filter. The light was transformed into a plane wave using a collimation lens. A linear polarizer and a quarter waveplate rotated by 45° were used to generate a left-hand circular polarized wave for illuminating the hologram. The incident wave was modulated by the hologram, and only the right-handed circular polarized wave was selected by using a quarter waveplate and a linear polarizer in combination; the quarter waveplate and linear polarizer were rotated by 45° and 90°, respectively. The diffraction wave was obtained using a telecentric lens in order to restrict the angular spectrum within a narrow range, since the modulation of holograms is sensitive to the diffraction angle.

### Replication of Si master to prepare polymer stamp

The UV-curable polyurethane acrylate imprint resin RM-311(Minuta Technology Co., Ltd, Korea) was used to replicate the angular nanoslits formed on the Si master. The imprint resin was poured on the Si wafer, and it was covered with a polyethylene terephthalate (PET) film. Then, pressure was applied to remove the trapped air and to fill the pattern. Next, UV curing was performed twice for 90 s each.

### Deposition of metals and SiO_2_ layers on polymer stamp

The metals and SiO_2_ layers were deposited on the polymer stamp by e-beam evaporation (Daeki Hi-Tech Co., Ltd, Korea) at a rate of 1 Å/s under high vacuum.

### Preparation of adhesive-coated glass substrate

N-[3-(trimethoxysilyl)propyl]ethylenediamine was used as the adhesive, and propane-1,3-diol and di(propylene glycol) methyl ether were used as the solvents. All the materials were purchased from Sigma-Aldrich. The components were mixed in volume fractions of 1%, 70%, and 29%, respectively. The adhesive solution was subjected to ultrasonication for 30 s and then spin-coated on the plasma-treated glass substrate at 5000 rpm for 60 s. The substrate was then baked for 60 s at 150 °C on a hot plate so that only the N-[3-(trimethoxysilyl)propyl]ethylenediamine adhesive remained^22^.

### Nanotransfer printing process

The adhesive-coated substrate and polymer stamp were placed on the roll-to-plate transfer system (Eastern Engineering, Korea), and pressure was applied on the polymer stamp with the four deposited layers.

### Fabrication of the angular nanoslit Si master

The angular nanoslit Si master was fabricated by e-beam lithography.

### Surface and cross-sectional image analysis

A focused ion beam (FIB) (Helios Nanolab, FEI, USA) and a transmission electron microscopy (TEM) (JEM-ARM200F, JEOL, Japan) were employed to obtain high-resolution surface and cross-sectional images.

### X-ray photoelectron spectroscopy (XPS) analysis

XPS (ThermoFisher Scientific, K-Alpha+, USA) was employed to determine the Au binding energies at the various interfaces. The electron energy analyzer is located in the normal direction to the sample surface; the angle between the normal to the sample surface and the analyzer is 0°, and an ion gun was used with 500 eV, 10 mA, and monoatomic Ar^+^.

## Supplementary information

Revised_Supplementary_information
