# Supplementary material for: Gold-Nanocluster-Assisted Nanotransfer Printing Method for Metasurface Hologram Fabrication
Source: Sci Rep. 2019 Feb 28;9:3051. doi: 10.1038/s41598-019-38891-2 (PMC6395669; doi:10.1038/s41598-019-38891-2)
Supplement: Supplementary file 1 — Revised_Supplementary_information [file 41598_2019_38891_MOESM1_ESM.docx]

**Supplementary Information**

**Gold-Nanocluster-Assisted Nanotransfer Printing Method for Metasurface Hologram Fabrication**

*Soon Hyoung Hwang,^a^ Jaebum Cho,^d^ Sohee Jeon,^b^ Hyeok-Jung Kang,^b^ Zhi-Jun Zhao,^b^ Sungjae Park,^c^ Yohan Lee,^d^ Jonghyun Lee,^c^ Mugeon Kim,^e^ Joonku Hahn,^e^ Byoungho Lee,^d^ Jun Ho Jeong,^b^* Hwi Kim,^c^* and Jae Ryoun Youn ^a^**

*^a^ Research Institute of Advanced Materials (RIAM), Department of Materials Science and Engineering, Seoul National University, Seoul, 08826 South Korea.*

*^b^ Nano-Convergence Mechanical Systems Research Division, Korea Institute of Machinery and Materials, Daejeon, 34103 South Korea.*

*^c^ Department of Electronics and Information Engineering, Korea University, Sejong, 30019 South Korea.*

*^d^ School of Electrical and Computer Engineering, Seoul National University, 1 Gwanak-ro, Seoul, 08826 Gwanak-gu, South Korea.*

*^e^ School of Electronics Engineering, Kyungpook National University, Daegu, 41566 South Korea.*

* Corresponding authors:

Dr. Jun Ho Jeong

Tel.: +82-42-868-7604; Fax: +82-42-868-7123

E-mail: jhjeong@kimm.re.kr

Prof. Hwi Kim

Tel.: +82-44-860-1428; Fax: +82-44-860-1048

E-mail: hwikim@korea.ac.kr

Prof. Jae Ryoun Youn

Tel.: +82-2-880-8326; Fax: +82-2-885-9671

E-mail: [jaeryoun@snu.ac.kr](mailto:jaeryoun@snu.ac.kr)


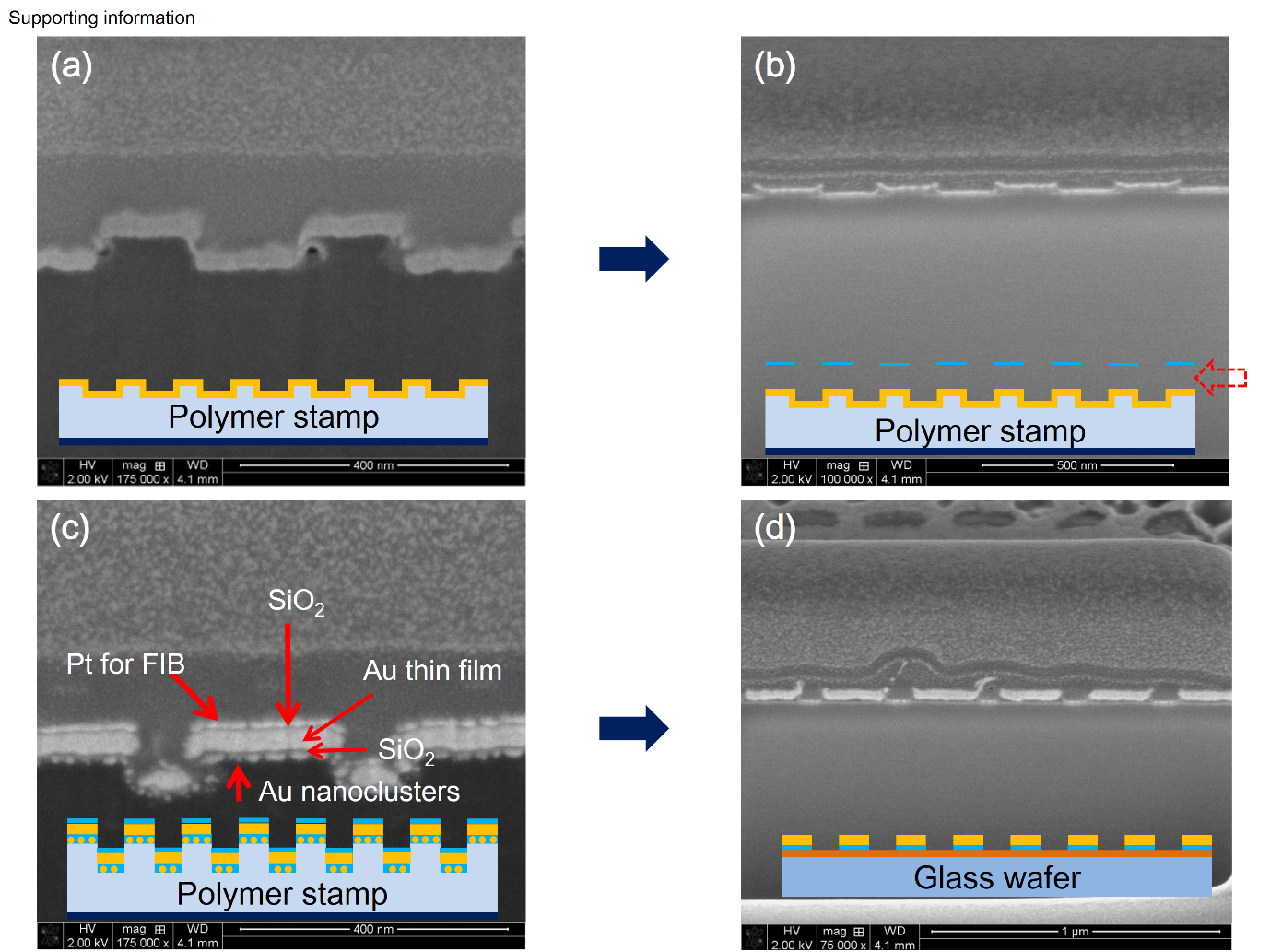


**Figure S1.** FIB cross-sectional images, an illustration of the deposited layers on a polymer stamp, and the materials transferred onto the substrate. (a) FIB cross-sectional image of the deposited 30‑nm Au thin film. (b) Only the SiO_2_ layer is transferred onto the glass substrate when two layers (Au and SiO_2_) are deposited on the polymer stamp. (c) FIB cross-sectional image of four layers of Au and SiO_2_ deposited on a polymer stamp. (d) Nanoslit metasurface fabricated by transferring SiO_2_ and Au layers onto an adhesive-coated 2-inch glass substrate.


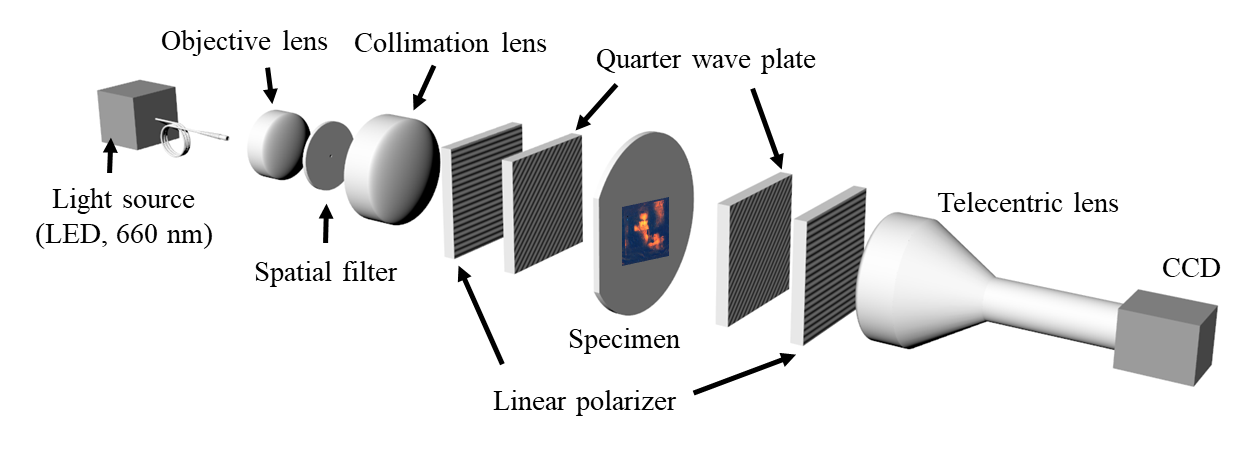


**Figure S2**. Setup used for analyzing the holograms.


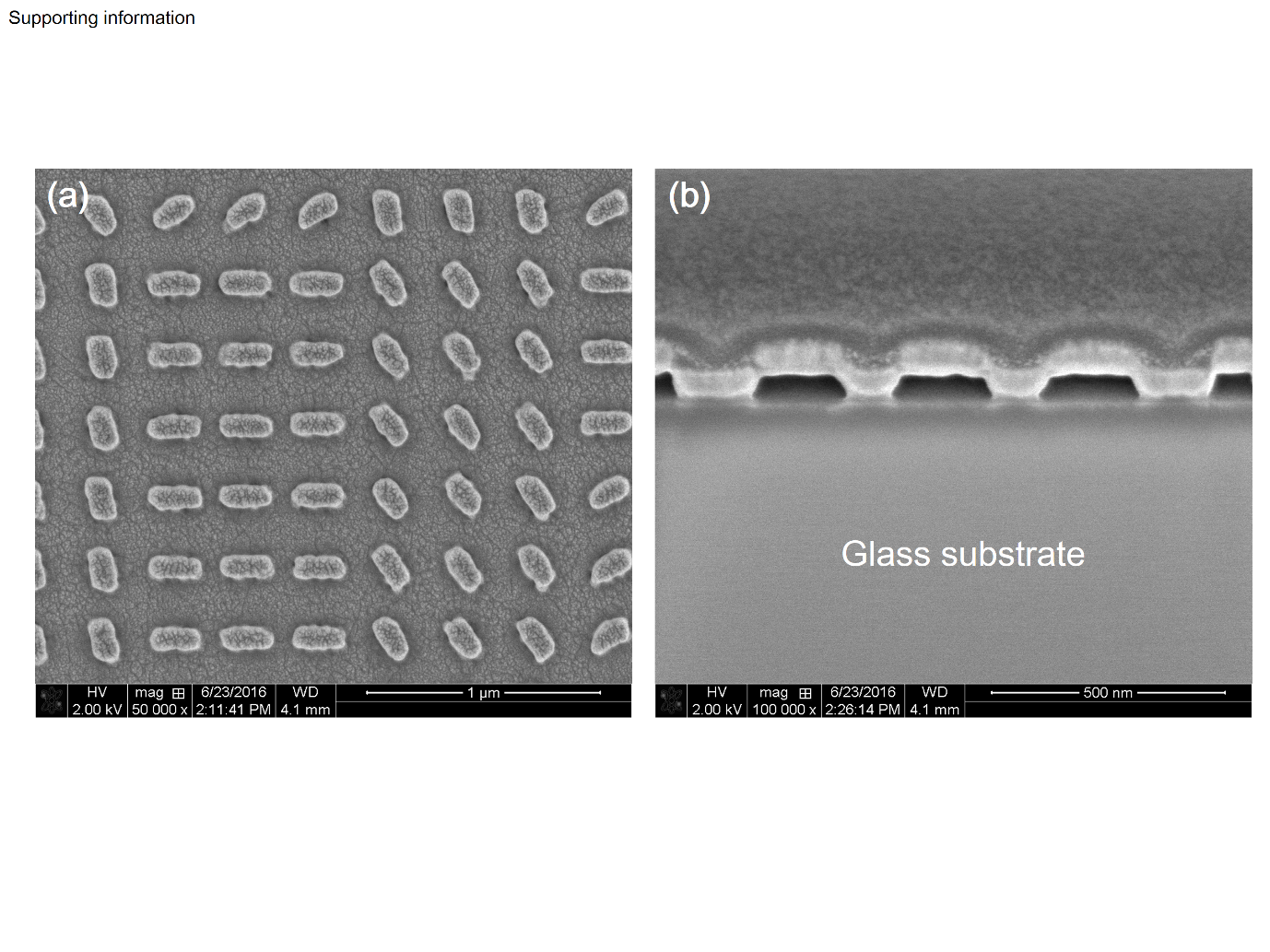


**Figure S3.** FIB images of the surface and cross-section of a glass substrate after nanotransfer printing of Ag and SiO_2_ layers deposited on a flexible polymer stamp.

**
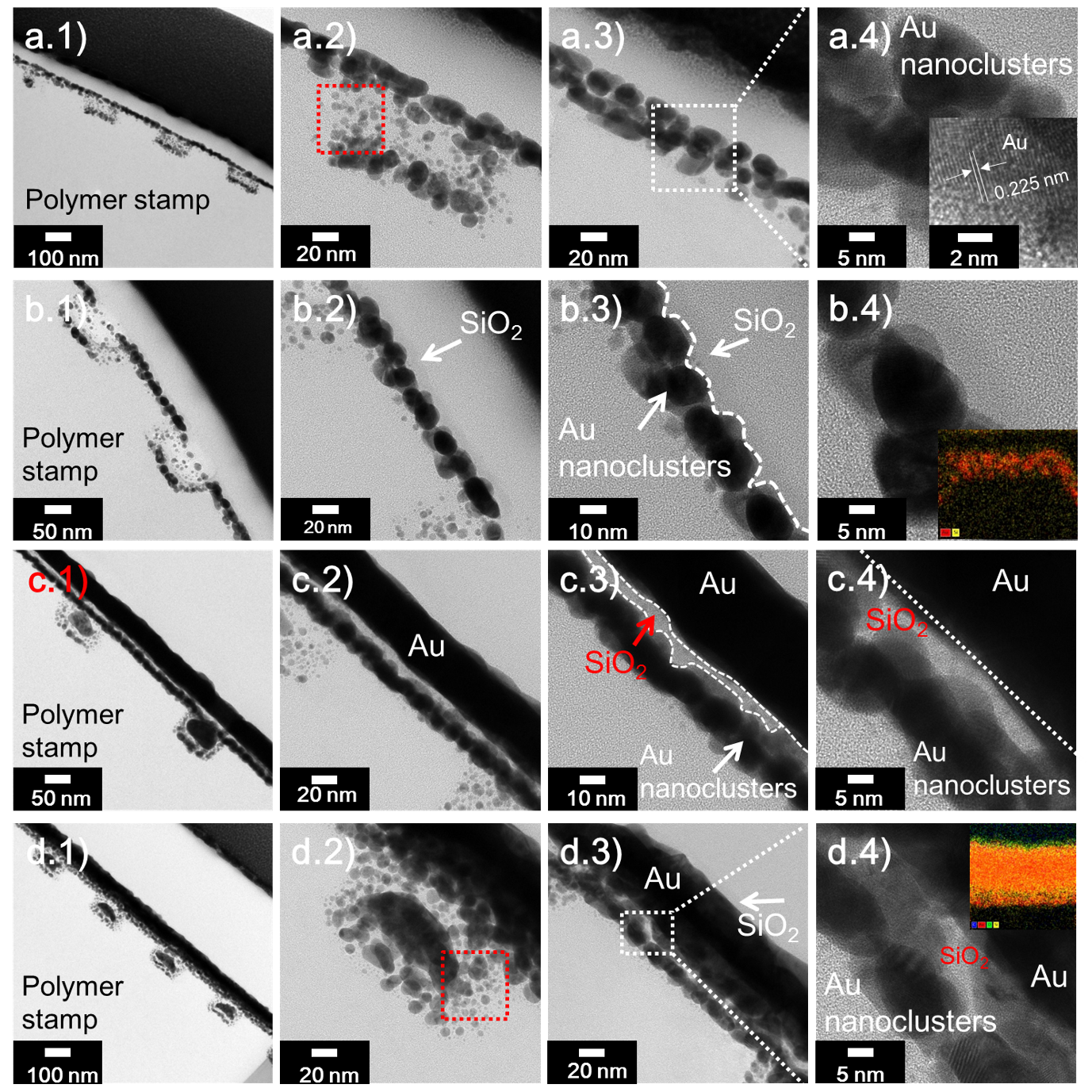
Figure S4.** TEM cross-sectional and EDS images of materials deposited on a polymer stamp at different magnifications. (a) TEM cross-sectional images of a polymer stamp with Au nanoclusters (inset shows the length of the Au lattice). (b) Polymer stamp with Au nanoclusters and an intermediate SiO_2_ layer (inset shows EDS images of Au and Si). (c) Polymer stamp with Au nanoclusters, intermediate SiO_2_ layer, and Au thin film (white dotted lines represent the boundaries of the materials). (d) Polymer stamp with Au nanoclusters, intermediate SiO_2_ layer, Au thin film, and outermost SiO_2_ layer (inset shows EDS images of Au, Si, O, and C).


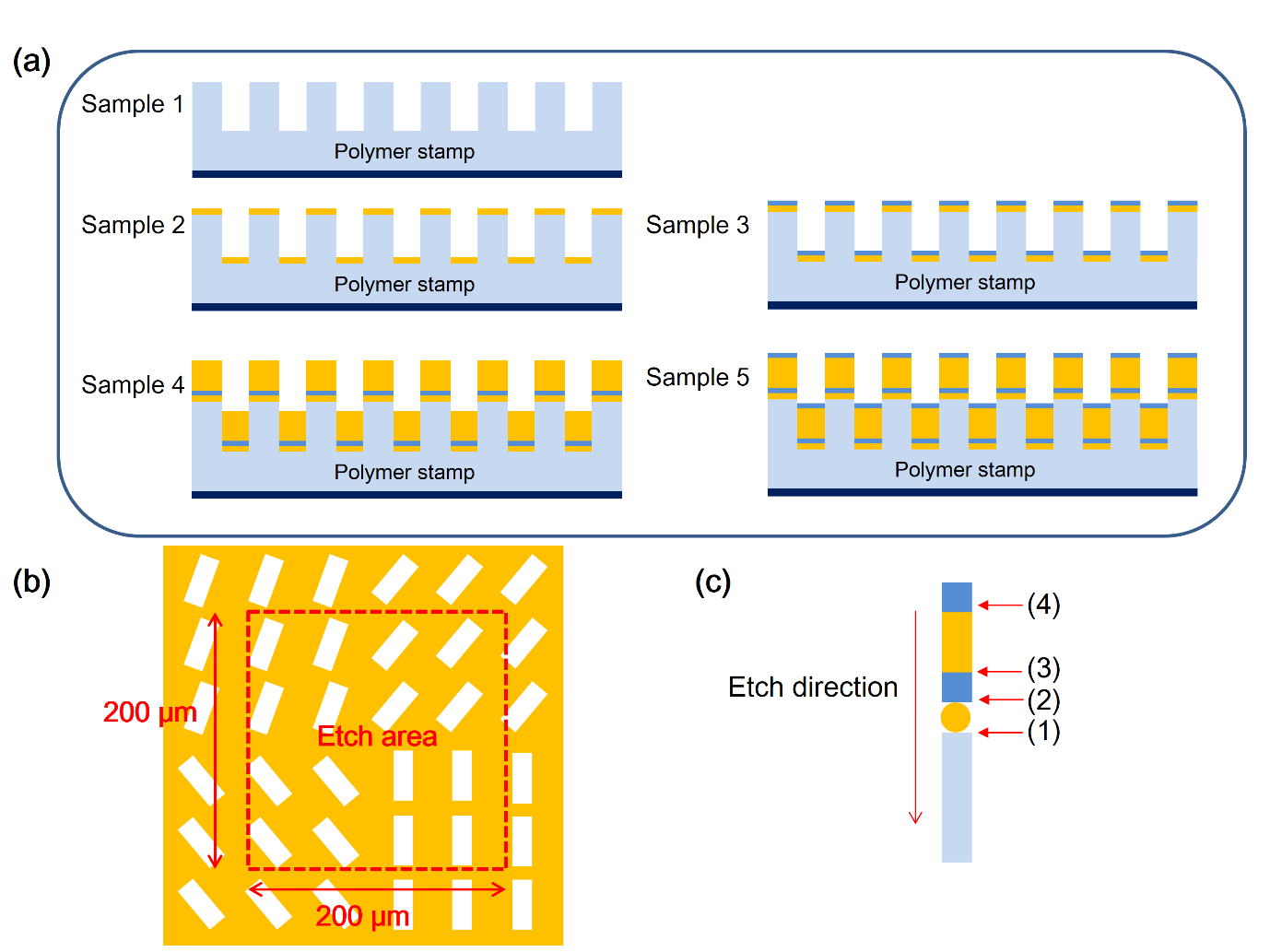


**
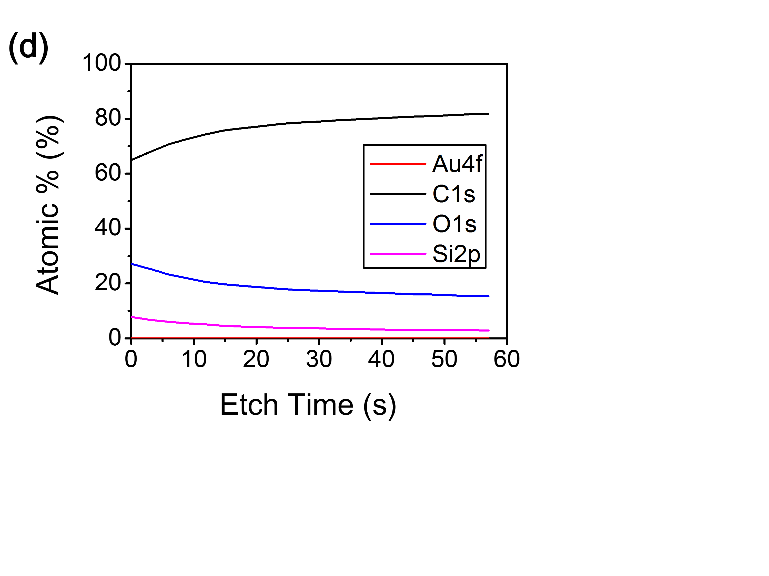
**

**Figure S5.** Illustration showing the preparation of samples used for XPS analysis and XPS spectra of the composition (at%) of a polymer stamp (a) Polymer stamp with alternately deposited Au and SiO_2_ layers. (b) Illustration of the 200 µm square etch area. (c) Illustration showing the etch direction and number labels of interfaces. (d) XPS spectra showing the composition (at%) of a polymer stamp with respect to etch time.


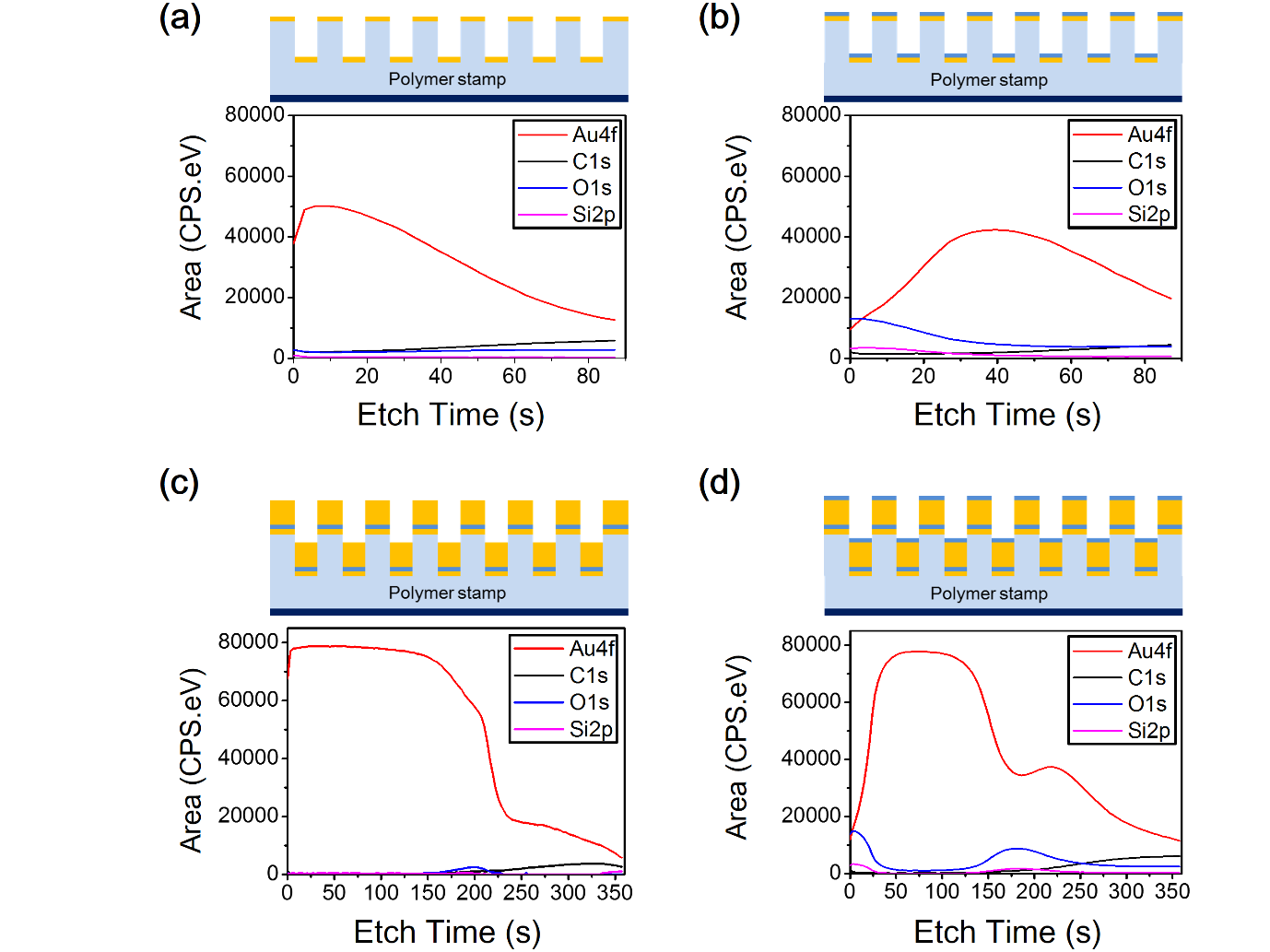


**Figure S6.** XPS spectra showing intensities of Au, C, O, and Si peaks as functions of the etch time and illustrations of the samples used for measurements.


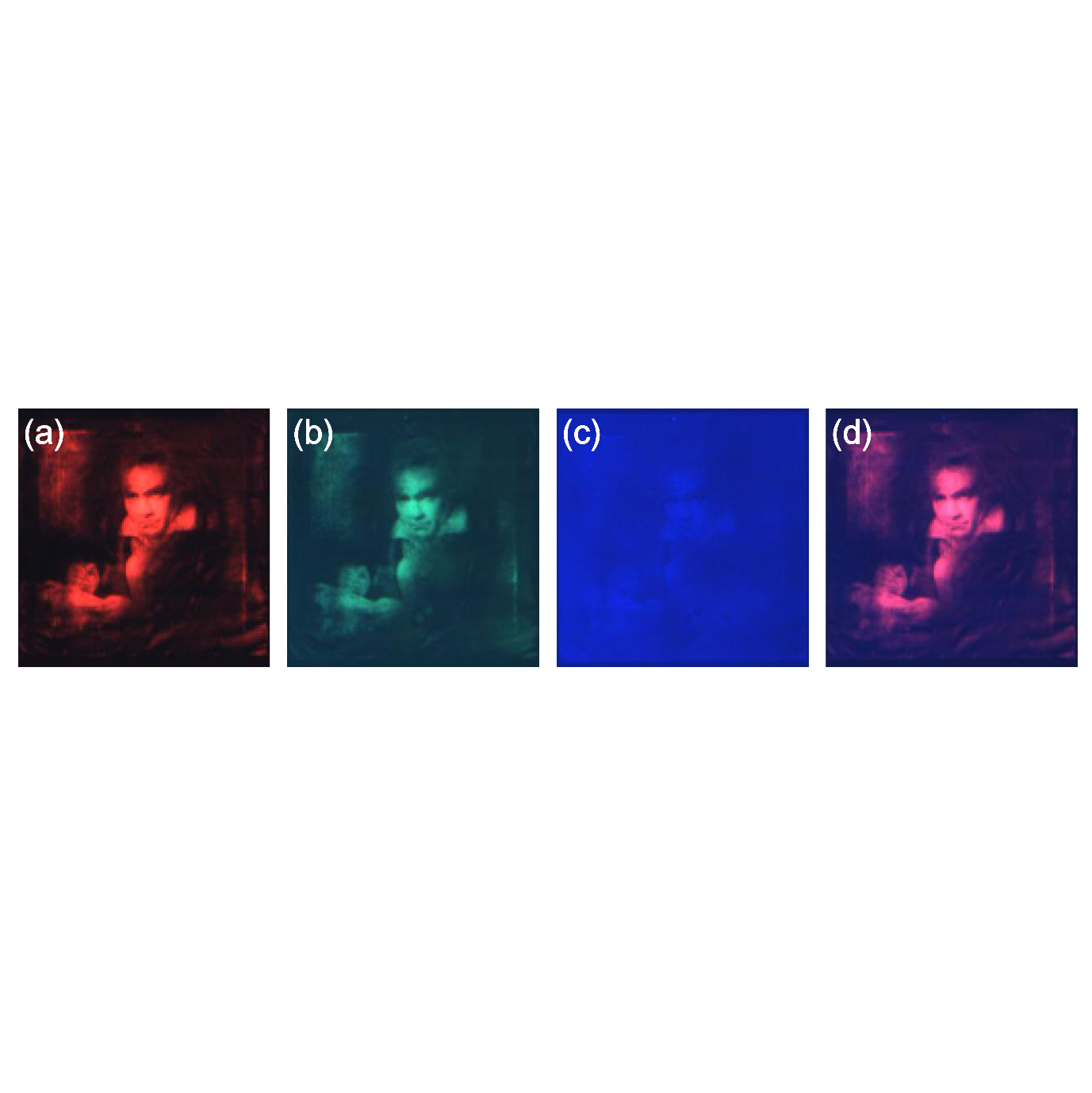


**Figure S7.** Measured metasurface hologram images of Au nanoslit metasurface using (a) red (660 nm) (b) green (530 nm), (c) blue (470 nm) and (d) white LED light sources.

**Fabrication of nanoslit- and nanorod-type metasurfaces using Au, Ag, and Al**

Ag and Al nanoslit metasurfaces were fabricated by depositing Ag and Al thin films instead of depositing the Au thin film between the intermediate and outermost SiO_2_ layers.

We successfully demonstrated the inverse design of the nanorod metasurface by replicating the silicon master once instead of twice. After preparing the polymer stamp, we first formed Au nanoclusters and then deposited intermediate SiO_2_, a metal thin film (Au, Ag, and Al), and the outermost SiO_2_ layer. For example, when the Ag nanorod metasurface was fabricated, we deposited 5-nm-thick Au to form nanoclusters on the surface of the nanorod-type polymer stamp. Next, a 5-nm-thick SiO_2_ layer, Ag thin film, and another 5-nm-thick SiO_2_ layer were deposited and transferred onto the adhesive-coated substrate. The Au nanoclusters prevent the connection between the materials on the top and in the trenches of the polymer stamp, enabling the successful fabrication of the Ag nanorod metasurface.


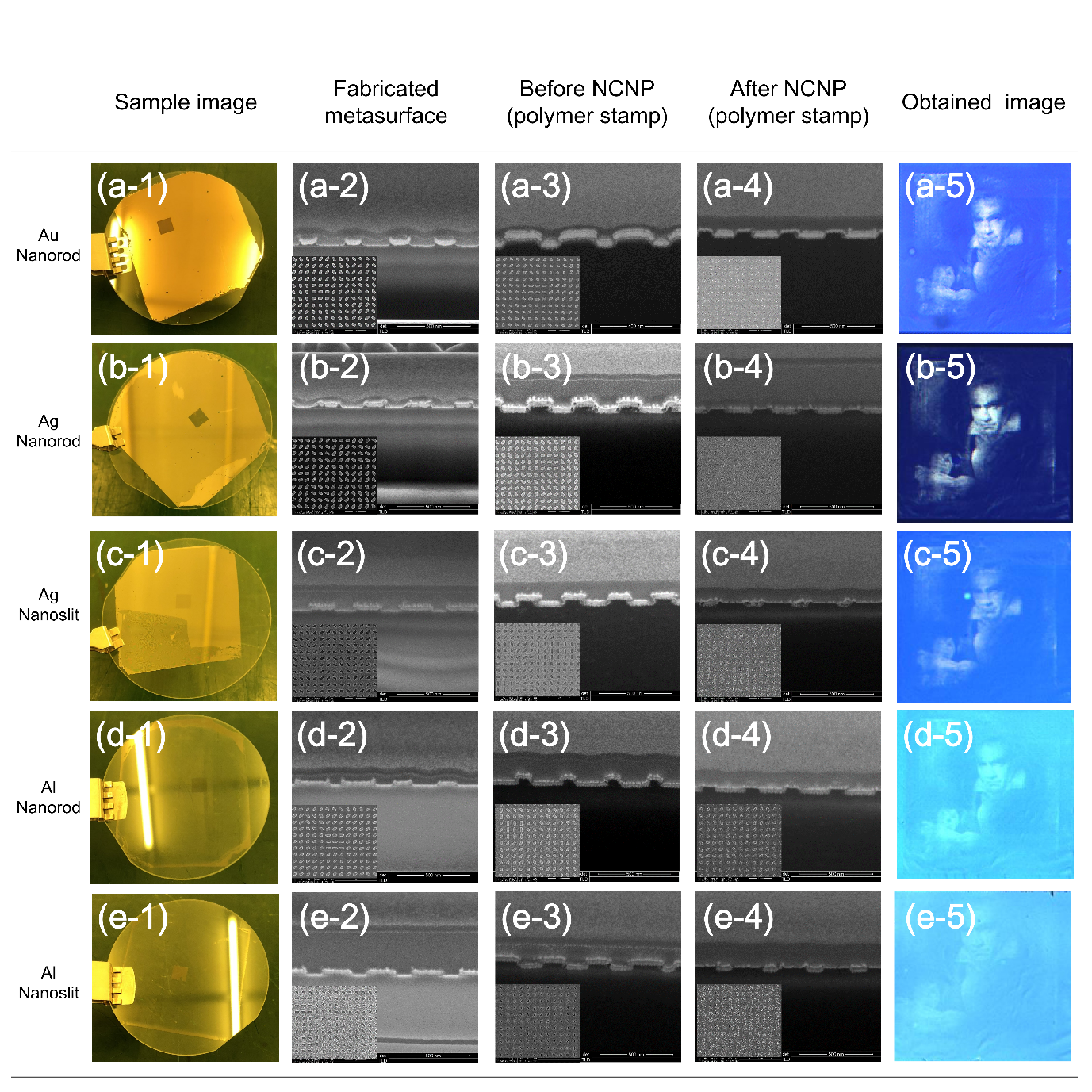


**Figure S8.** Images of the fabricated angular nanorod and nanoslit metasurface. (a-1) Fabricated Au nanorod metasurface. Surface and cross-sectional FIB images of (a-2) the fabricated Au nanorod metasurface, (a-3) the polymer stamp with deposited materials, and (a-4) the polymer stamp after NCNP method. (a-5) Experimentally obtained hologram image from the Au nanorod metasurface with white LED light source. (b-1) Fabricated Ag nanorod metasurface. Surface and cross-sectional FIB images of (b-2) the fabricated Ag nanorod metasurface, (b-3) the polymer stamp with deposited materials, (b-4) and the polymer stamp after NCNP method (b-5). Experimentally obtained hologram image from the Ag nanorod metasurface with white LED light source. (c-1) Fabricated Ag nanoslit metasurface. Surface and cross-sectional FIB images (c-2) of the fabricated Ag nanoslit metasurface, (c-3) the polymer stamp with deposited materials, and (c-4) the polymer stamp after NCNP method. (c-5) Experimentally obtained hologram image from the Ag nanoslit metasurface with white LED light source. (d-1) Fabricated Al nanorod metasurface. Surface and cross-sectional FIB images of (d-2) the fabricated Al nanorod metasurface, (d-3) of the polymer stamp with deposited materials, and (d-4) of the polymer stamp after NCNP method. (d-5) Experimentally obtained hologram image from the Al nanorod metasurface with white LED light source. (e-1) Fabricated Al nanoslit metasurface. Surface and cross-sectional FIB images of (e-2) the fabricated Al nanoslit metasurface, (e-3) the polymer stamp with deposited materials, (e-4) and the polymer stamp after NCNP method (e-5). Experimentally obtained hologram image from the Al nanoslit metasurface with white LED light source.
